# Digital Mental Health Coaching in Clinically Diverse Populations: Controlled Engagement and Outcomes Study

**DOI:** 10.2196/71346

**Published:** 2025-10-27

**Authors:** Alison Pickover, Sarah Adler

**Affiliations:** 1 Wave Life, Inc., 700 El Camino Real, Suite 120, Menlo Park, CA, 94025, United States, 1 9176016840, 1 5303991880

**Keywords:** coaching, digital health, mental health, transdiagnostic, outcome, depression, anxiety, stress

## Abstract

**Background:**

Digital coaching programs, offering virtually delivered mental health care by coaches and companion apps, are an increasingly popular care model designed to increase accessibility and reduce strain on traditional mental health care systems. Initial studies suggest these programs can produce a range of positive mental health outcomes; however, methodological limitations and a focus on homogeneous, subclinical populations have constrained conclusions about their effectiveness, especially in diverse and clinically severe samples.

**Objective:**

This study aimed to evaluate the impact of an evidence-based digital mental health coaching program in a clinically and demographically diverse sample. The study compared engagement with app-based content and changes in depression, anxiety, and stress symptoms, over the course of 1 month, among users who received coaching versus those who used the app alone (controls).

**Methods:**

Program users (N=64) were categorized as coaching users (attending at least 1 session) or controls (app-only users). Depression, anxiety, and stress symptoms were assessed using the Depression Anxiety Stress Scale-21 at baseline and after 30 days. Engagement with app content was also measured. Between-group differences were analyzed using *t* tests and mixed multivariate analysis of covariance models, with follow-up sensitivity analysis of covariance analyses (controlling for age).

**Results:**

Participants were diverse in terms of demographics and clinical severity, with half reporting severe to extremely severe depression and nearly half reporting severe to extremely severe anxiety or stress at baseline. A repeated-measures multivariate analysis of covariance revealed a significant group-by-time interaction (*P*=.02), indicating greater symptom reduction among coaching users, primarily driven by changes in anxiety and stress. Follow-up analyses of covariance exploring symptom-specific patterns, excluding participants with subclinical baseline symptoms, yielded significant group-by-time interactions across depression (*P*=.04), anxiety (*P*=.003), and stress (*P*=.03). Engagement with app-based content did not significantly differ between the groups (*P*=.20), suggesting coaching’s effectiveness was not contingent on differential app usage.

**Conclusions:**

This study demonstrates that digital mental health coaching can significantly improve clinical outcomes, even in diverse and clinically severe populations. These findings challenge the notion that coaching is only effective for subclinical or high-functioning individuals and highlight its potential to extend the reach of mental health care to underserved communities.

## Introduction

Mental health issues are common, with 1 in 5 adults experiencing mental health difficulties each year [1]. These issues are a significant source of disability, outranking physical health conditions (eg, cardiovascular and circulatory diseases) in terms of global disease burden [2]. They also frequently co-occur with physical health concerns and contribute to poorer health outcomes. Mental health issues affect workplace productivity and are key contributors to absenteeism and turnover. Global estimates of mental health–related productivity loss exceed US $1 trillion annually [3].

Given the individual, social, and economic ramifications of poor mental health, increasing access to effective mental health care services is a crucial priority. Currently, more than half of those with mental illness do not receive treatment, largely due to the dearth of licensed providers available to treat mental health conditions [4,5]. In the US, where 150 million adults seek services annually [6,7], only 1.2 million behavioral health providers are available to provide care [8]. Half of Americans live in a mental health workforce shortage area [4,9], and workforce projections suggest this condition will only worsen with time [10].

Against this backdrop, there has arisen substantial innovation in approaches to mental health care delivery. Key developments include the provision of care virtually via telehealth, the inclusion of digital health tools and content, and service delivery by lay health workers like coaches. These shifts are notable for the following reasons: in the midst of a provider shortage marked by geographic disparity, they increase access to care (irrespective of location); and, without sacrificing the “human element,” they reduce the cost of providing health care. These approaches are also valuable levers for improving treatment retention [11] and for achieving health equity among vulnerable and underserved communities [12,13].

Digital coaching programs, offering virtually delivered care by coaches and companion apps, are an increasingly popular care model or care model component that seeks to increase care accessibility and reduce strain on mental health care systems. In such programs, services are provided by coaches who have received mental health care training or credentialing, but not necessarily licensure. These coaches serve two valuable and complementary functions: (1) helping individuals set and achieve goals via supportive accountability and skill-building and (2) improving user engagement with app-based digital resources to ensure maximum benefit [14,15]. Preliminary studies suggest that digital coaching programs can produce a range of positive mental health outcomes in select populations. For instance, in employee samples, digital coaching programs are associated with within-group improvements in well-being [16,17], emotional intelligence [16], self-efficacy, emotion regulation, social connectedness [18], and stress [17]. In at least 1 study, coaching has been shown to impact employees’ levels of depression and anxiety as well [19].

Although initial data regarding the effectiveness of digital coaching programs look promising, critical methodological issues limit enthusiasm. First, available studies lack a control condition, and thus, it is not clear that coaching is responsible for observed improvements. Second, many studies, by virtue of evaluating employee benefits programs, focus on homogeneous samples with subclinical or mild to moderate symptomatology. As such, it remains unclear whether these programs yield positive outcomes in more representative and diverse samples. Research that improves upon these limitations is necessary to realize the true potential of coaching in driving clinical outcomes and reducing care disparities.

In this study, we sought to evaluate the impact of a digital coaching program in a clinically and demographically diverse sample. Specifically, we evaluated differences in content engagement and reductions in depression, anxiety, and stress over the course of a month of engagement with the digital coaching program Wave. To assess whether coaching was a key driver of outcomes, we compared engagement and symptom reductions among coaching users to Wave users who downloaded Wave’s app (developed by Wave Life, Inc.) but did not engage in coaching. Based on previous literature [20] and the coach’s hypothesized role in fostering supportive accountability [15], we predicted greater content engagement by coaching users than their counterparts. Given some evidence that coaching can reduce clinical symptoms [19], we predicted reductions in depression, anxiety, and stress among users who engaged in coaching and predicted that these reductions would be steeper than any reductions observed among users who did not engage in coaching.

## Methods

### Study Design and Participants

Participants were a cohort of Wave users (N=64) who created accounts between April 2023 and May 2024. Account creation was restricted to individuals 18 years of age or older who resided in the United States. Participants either had access to Wave as an employment benefit (11/64, 17%) or accessed Wave independently by downloading Wave’s app in the Apple or Google Play Stores (53/64, 83%); independent users typically learned about Wave through targeted social media advertisements (eg, Facebook [Meta], Instagram [Meta], and LinkedIn [LinkedIn Corporation]) or through personal recommendations and word-of-mouth referrals. Wave users were included in the study if they provided symptom data at at least 2 timepoints, within a 45-day interval, during their course of using Wave. Users were prompted to complete symptom measures in advance of their first coaching session or at the end of account creation, and they received subsequent prompts to complete symptom measures at 30-day intervals. Use of Wave’s app and coaching services was voluntary, and users had the option to cease engagement at any time. Measure completion, app use, and coaching took place remotely.

### Measures

#### Demographic Characteristics

Date of birth was self-reported at the time of account creation and transformed to age. In addition, users had the opportunity to fill out an in-app demographic survey assessing gender, race and ethnicity, and relationship status. Completion of the demographic survey was optional.

#### Depression, Anxiety, and Stress

Users completed the Depression Anxiety Stress Scale-21 (short version; DASS-21) [21] twice via an in-app, self-report questionnaire. The DASS-21 is a well-validated [22-24] measure comprising 3 scales, each with 7 items. Item responses were summed to create continuous scores of depression, anxiety, and stress symptom severity; higher scores indicated greater severity. In addition, using established thresholds [21], the DASS-21 was used to classify depression, anxiety, and stress symptoms as normal, mild, moderate, severe, or extremely severe, allowing for categorical interpretation of symptom severity.

#### Content Engagement

Content engagement was measured as the number of in-app resources (short-form written, audio, and video content) viewed by a user during their time on the platform. Due to evolving methods of tracking user engagement with content, data were only available for 25 of 38 (66%) coaching users; however, content engagement data were available for 100% of controls. Available data regarding content engagement was recorded passively through the app.

### Intervention

Users received one-on-one behavioral health coaching and access to Wave’s mobile app. Users interested in coaching services were given the opportunity to schedule sessions upon account creation or at a later date. Sessions could be scheduled the same day or for the future. Coaching services were provided via remote video visits. Typically, sessions occurred weekly and lasted 30 minutes; however, users could request alternative session intervals or longer sessions. Coaches helped users create customized care programs based on their unique needs; they assisted users in defining and achieving wellness goals via psychoeducation and skills acquisition. Coaches used a transdiagnostic approach to case conceptualization and incorporated skills associated with evidence-based therapies such as acceptance and commitment therapy and cognitive behavioral therapy. Coaches had access to users’ DASS-21 responses, which they reviewed with users in session, to help focus sessions and as a means of tracking progress over time.

All Wave coaches were bachelor’s and master’s-level practitioners who received certification from the National Board for Health and Wellness Coaching. This certification is achieved by completing an approved training program, acquiring 50 posttraining practice sessions, and passing an exam cosponsored by the National Board of Medical Examiners. Wave provided additional coach training covering foundational practice components (eg, rapport, supportive accountability, and cultural humility), Wave’s proprietary technology and app, mental health first aid, motivational interviewing, case conceptualization, risk assessment and management, and use of quantitative data in measurement-based care. Training included both didactics and role-play exercises. While providing services at Wave, coaches attended a weekly meeting for case review and consultation.

All Wave users, regardless of their coaching status, received access to Wave’s app. The app provided a variety of tools and content designed to support users in managing their health and emotional well-being. Content included short-form written, audio, and video content and spanned psychoeducation, guided practice, and skill-building. All content was reviewed for accuracy by licensed clinical psychologists.

### Analytic Plan

To compare progress over time, users were separated into 2 groups: coaching users (n=36) attended at least 1 coaching session between their first and second DASS-21 completions; controls (n=28) attended zero sessions in that interval.

Data were checked for outliers, skew, and kurtosis prior to all analyses; age and content engagement were shown to be (positively) skewed. To identify potential confounds, *t* tests and chi-square tests were used to assess between-group differences in demographic characteristics (age was log-transformed), baseline clinical symptom severity, and content engagement (square root-transformed).

A repeated-measures multivariate analysis of covariance (MANCOVA) was used to assess the impact of coaching on symptom change over time, controlling for age. MANCOVA was selected to account for the high correlation among the 3 dependent variables (depression, anxiety, and stress, continuously assessed), reduce the risk of type I error associated with multiple univariate tests, and allow for the evaluation of an overall multivariate interaction effect. Prior to this analysis, the data were checked for multivariate normality. Based on visual inspection of the data and a significant Shapiro-Wilk test, follow-up depression scores were square-root transformed for this analysis. Multivariate outliers, assessed using Mahalanobis distance, were not observed. Inspection of scatterplot matrices revealed linear relationships between the dependent variables, and bivariate correlations (*r*<.90) ruled out multicollinearity concerns. Box M test (*P*=.01) was used to confirm homogeneity of covariance matrices.

To better understand symptom-specific intervention effects among participants with elevated symptoms, follow-up analyses of covariance (ANCOVAs) were conducted as sensitivity analyses, excluding participants who scored in the “normal” range at baseline on the corresponding DASS-21 scale. For example, the depression-specific ANCOVA was conducted on data drawn from users who initially scored in the mild, moderate, severe, or extremely severe ranges on the DASS-21 depression scale. This approach was used to reduce potential floor effects and to ensure sufficient variability for detecting change among individuals with room for improvement in each domain.

### Ethical Considerations

All data were deidentified prior to analysis, and the use of human participant data was reviewed and determined to be exempt by an institutional review board (Advarra Pro00079304). Users provided consent for the use of their deidentified data for research purposes by accepting the platform’s Privacy Policy at account creation. Users were not compensated for their use of Wave or completion of measures.

## Results

### Demographic Characteristics and Initial Clinical Severity

Sample descriptive statistics are presented in Table 1. Table 2 summarizes symptom severity among the sample.

Participants were diverse in terms of their demography and clinical presentation. Half of the sample reported severe to extremely severe depression, and nearly half reported severe to extremely severe anxiety or stress. On average, coaching users were older than controls (mean_coaching_ 35.78, SD 12.10 years; mean_control_ 28.46*,* SD 9.10 years; *t*_62_=–2.78; *P=*.007). Chi-square tests showed that coaching and control groups did not significantly differ in gender (*P=*.09), race and ethnicity (*P=*.38), or relationship status (*P=*.63). Chi-square tests showed that the distribution of participants across clinical severity categories (mild to extremely severe) did not differ significantly between coaching users and controls; this held true for depression (*P=*.23), anxiety (*P=*.10), and stress (*P=*.57) severity. Likewise, no significant baseline differences in symptom severity were found when assessing severity continuously (*P=*.62, *P=*.43, and *P=*.10, for depression, anxiety, and stress, respectively). The average time between DASS-21 responses was 34.77 (SD 5.33) days.

**Table 1.** Sample descriptive statistics.^a^

Characteristics	Full sample (N=64)	Coaching users (n=36)	Control group (n=28)
Age (years), mean (SD)	32.58 (11.41)	35.78 (12.10)	28.46 (9.10)
Gender, n (%)			
Woman	36 (56.3)	16 (44.4)	20 (71.4)
Man	13 (20.3)	11 (30.6)	2 (7.1)
Other not listed	3 (4.7)	2 (5.6)	1 (3.6)
Agender	1 (1.6)	1 (2.8)	0 (0)
Gender nonconforming	1 (1.6)	0 (0)	1 (3.6)
Prefer not to answer or no response	10 (15.6)	6 (16.6)	4 (14.3)
Race and ethnicity^b^, n (%)			
White	35 (54.7)	20 (55.6)	14 (50)
Black or African American	11 (17.2)	5 (13.9)	6 (21.4)
Asian	9 (14.1)	5 (13.9)	4 (14.3)
Hispanic or Latino	10 (15.6)	2 (5.6)	7 (25)
Native American or Alaska Native	2 (3.1)	1 (2.8)	1 (3.6)
Prefer not to answer or no response	5 (7.8)	3 (8.3)	2 (7.1)
Relationship status, n (%)			
Single	18 (28.1)	8 (22.2)	10 (35.7)
Partnered	19 (29.7)	7 (19.4)	12 (42.9)
Married	8 (12.5)	5 (13.9)	3 (10.7)
Separated or divorced	3 (4.7)	2 (5.6)	1 (3.6)
Prefer not to answer or no response	16 (25)	14 (38.9)	2 (7.1)

a Demographic characteristics of a cohort of US adult users of the Wave digital mental health platform, including users who received remote coaching (n=36) and controls who only used the app (n=28), are listed. Data were collected remotely between April 2023 and May 2024. Users self-reported date of birth (transformed to age) upon account creation and provided other demographic data via an optional, in-app questionnaire.

b Users could select more than 1 response option for race and ethnicity.

**Table 2.** Symptom severity.^a^

Symptoms	Baseline, mean (SD)		30‐45 days later, mean (SD)	
	Coaching	Control	Coaching	Control
Depression	21.22 (12)	22.64 (10.58)	14.22 (10.6)	20.07 (11.46)
Anxiety	13.78 (6.25)	15.36 (8.80)	9.00 (6.11)	15.21 (8.85)
Stress	20.56 (8.63)	23.93 (7.78)	15.89 (8.78)	22.57 (8.43)

a Depression Anxiety Stress Scale-21 (DASS-21) scores at baseline and 30‐45 days later among a cohort of the US adult users of the Wave digital mental health platform, including users who received remote coaching (n=36) and controls who only used the app (n=28), are listed. Data were collected remotely via in-app measures between April 2023 and May 2024.

### Engagement

Coaching users attended a median of 4 (IQR 4-5) sessions between first and second DASS-21 measurements. On average, they engaged more heavily with content (median 21, IQR 8-38 pieces of content) than their control counterparts (median 11, IQR 3.25-29.75 pieces of content) during this period; however, this difference did not reach statistical significance (*t*_51_=–1.30; *P*=.20).

### Clinical Improvement

MANCOVA revealed a significant group-by-time interaction (Wilks λ=0.85, *F*_3,59_=3.55, partial η²=0.153; *P*=.02), indicating that symptom changes over time varied by condition across the combined outcomes of depression, anxiety, and stress. Follow-up univariate tests showed a significant group-by-time interaction for anxiety (*P*=.004) and stress (*P*=.04). No significant interaction effect (=.67) or main effect of time (=.56) was observed for depression.

### Sensitivity Analyses

**Figure 1.**
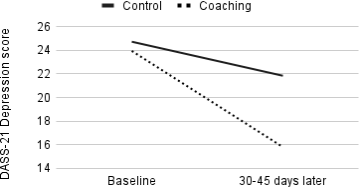
Temporal change in depression symptoms, as measured by the DASS-21, among the US adult users of a digital mental health platform (Wave) who received remote coaching versus those who used the app only. Data were collected between April 2023 and May 2024. ANCOVA revealed a significant group-by-time interaction (*P*=.04, medium effect size): coaching users with elevated baseline depression symptoms showed significantly greater reductions in symptoms compared to controls who did not engage in coaching. ANCOVA: analysis of covariance; DASS-21: Depression Anxiety Stress Scale-21.

In the sensitivity analyses, significant group-by-time interactions were observed for depression, anxiety, and stress, with coaching users faring better over time than their control group counterparts (Figures1^3^). In the cases of depression (Wilks λ=0.92, *F*_1,53_=4.65, partial η²=0.081; *P*=.04) and stress (Wilks λ=0.91, *F*_1,48_=4.95, partial η²=0.093; *P*=.03), ANCOVAs revealed statistically significant symptom improvement following coaching with moderate effect sizes. In the case of anxiety, statistically significant improvement following coaching was associated with a large effect size (Wilks λ=0.85, *F*_1,52_=9.55, partial η²=0.155; *P*=.003).

**Figure 2.**
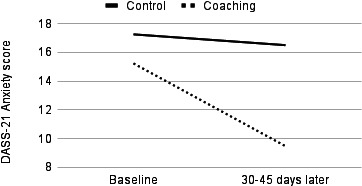
Temporal change in anxiety symptoms, as measured by the DASS-21, among the US adult users of a digital mental health platform (Wave) who received remote coaching versus those who used the app only. Data were collected between April 2023 and May 2024. ANCOVA revealed a significant group-by-time interaction (*P*=.003, large effect size): coaching users with elevated baseline anxiety symptoms showed significantly greater reductions in symptoms compared to controls who did not engage in coaching. ANCOVA: analysis of covariance; DASS-21: Depression Anxiety Stress Scale-21.

**Figure 3.**
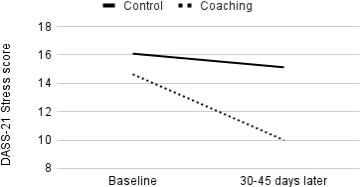
Temporal change in stress symptoms, as measured by the DASS-21, among the US adult users of a digital mental health platform (Wave) who received remote coaching versus those who used the app only. Data were collected between April 2023 and May 2024. ANCOVA revealed a significant group-by-time interaction (*P*=.03, medium effect size): coaching users with elevated baseline stress symptoms showed significantly greater reductions in symptoms compared to controls who did not engage in coaching. ANCOVA: analysis of covariance; DASS-21: Depression Anxiety Stress Scale-21.

## Discussion

### Principal Findings

In this study, coaching was associated with significant symptom improvement, particularly for individuals with clinically elevated symptoms of depression, anxiety, and stress. These results replicate and extend those of other studies, wherein, over the course of coaching, well-being and the ability to manage difficult emotions improved [16-18]. They lend credence to a sparse evidence base on coaching’s impact on anxiety [19] and coalesce with a larger literature examining “guided,” internet-based interventions for depression [25]. They suggest that coaching is a potent factor in symptom reduction, producing gains that exceed those gleaned from a fully self-guided experience. By incorporating a control group, rather than solely examining within-group changes, this study provides stronger evidence than has been previously presented regarding the causal role of coaching in temporal mental health improvements.

Notably, this study included a sample diverse in its demographic and clinical composition. More than half of the participants who sought coaching reported severe or extremely severe depression, anxiety, or stress. This marks a departure from most other studies, in which demographic data were not disclosed, and individuals with severe depression or anxiety were excluded [16,17,19,26]. Such exclusion criteria reflect a tacit, but untested, notion that coaching is not appropriate for high-acuity individuals. This notion may stem from coaching’s early roots as a workplace intervention designed to reduce stress in subclinical populations [27]. However, the results of this study challenge conventional notions regarding coaching’s narrow applicability. Instead, they suggest that, with training, lay health workers like coaches can effectively deliver evidence-based interventions that produce swift improvements in mental health across the clinical severity continuum. Given the potential impact of coaching on extending the reach of traditional health care systems, especially to vulnerable communities, it is critical that more studies assess coaching’s effectiveness among heterogeneous samples.

Despite group differences in symptom trajectories, coaching users and controls did not significantly differ in their engagement with content. This result is somewhat surprising given the posited role of coaches in increasing engagement with mental health apps [14]. However, while coaching may not have affected the quantity of engagement, it is possible that it impacted the quality, with downstream effects on symptomatology. For instance, among coaching users, content may have reinforced or extended what was discussed in coaching sessions, whereas among controls, content may have been encountered in a more exploratory or passive fashion that was less impactful. How best to define engagement with digital mental health interventions remains an open issue [20], and more research is needed to understand the interplay between coaching and the use of digital resources.

### Limitations

This study was limited in notable ways. The sample was small, and demographic data and content engagement data were not available for the full sample. Symptom severity was assessed by self-report measures roughly a month after initiating use of Wave; long-term data were not sufficiently available to assess maintenance of gains over time. Users were not randomly assigned to groups, and it is plausible that those who engaged in coaching were more intrinsically motivated, which could have affected the measured outcomes. Although we found that, despite nonrandom assignment, coaching users and controls were similar in terms of demographic characteristics assessed (when such data were available), and in initial symptom severity, other dimensions might differentiate these groups that could account for their different symptom trajectories. For instance, use of other mental health services or apps might have varied between the groups and could have contributed to the observed outcomes. As these variables were not assessed, they could not be accounted for in our analyses. Accordingly, larger, randomized clinical trials are essential to further establish the effectiveness of digital coaching programs.

### Conclusions

Despite these limitations, this study reflects an important step toward evaluating the effectiveness of novel approaches to mental health care delivery. It offers promising findings for disseminating evidence-based practice and reducing strain on an overburdened health care system. Research studies should continue to evaluate coaching’s impact on outcomes, including engagement and retention with digital mental health interventions, and long-term symptom reductions. These studies should take care to include diverse samples to provide a fuller understanding of coaching’s value in reducing care disparities.

## Abbreviations

ANCOVA: analysis of covariance
DASS-21: Depression Anxiety Stress Scale-21
MANCOVA: multivariate analysis of covariance
